# Antibacterial and Remineralizing Fillers in Experimental Orthodontic Adhesives

**DOI:** 10.3390/ma12040652

**Published:** 2019-02-21

**Authors:** Carolina Jung Ferreira, Vicente Castelo Branco Leitune, Gabriela de Souza Balbinot, Felipe Weidenbach Degrazia, Marianna Arakelyan, Salvatore Sauro, Fabricio Mezzomo Collares

**Affiliations:** 1 Dental Materials Laboratory, School of Dentistry, Universidade Federal do Rio Grande do Sul, Porto Alegre 90035-003, Brazil; carolinajungferreira@yahoo.com.br (C.J.F.); vicente.leitune@ufrgs.br (V.C.B.L.); gabriela.balbinot@ufrgs.br (G.d.S.B.); fdegrazia@hotmail.com (F.W.D.); 2 Department of Therapеutic Dentistry Sechenov University, Mozhaisky Val, 11 119435 Moscow, Russia; maristom87@inbox.ru; 3 BioMat-Dental Biomaterials Laboratory, Faculty of Health Sciences, University CEU-Cardenal Herrera, 46115 Valencia, Spain; salvatore.sauro@uchceu.es; 4 Dental Materials Laboratory, School of Dentistry, Universidade Federal do Rio Grande do Sul, Porto Alegre 90035-003, Brazil

**Keywords:** adhesive, orthodontic, mineral deposition, anti-bacterial agents, quaternary ammonium compounds, boron nitride

## Abstract

Orthodontic adhesives with antimicrobial and remineralizing properties may be an alternative to control white spot lesions around brackets. The aim of this study is to develop an experimental orthodontic adhesive containing boron nitride nanotubes (BNNT) and alkyl trimethyl ammonium bromide (ATAB). Methacrylate (BisGMA and TEGDMA) monomers were used to formulate the adhesives. Four experimental groups were produced with the addition of 0.1 wt.% BNNT (G_BNNT_); 0.1 wt.% ATAB (G_ATAB_); and 0.2 wt.% BNNT with ATAB (G_BNNT/ATAB_); in the control group, no fillers were added (G_Ctrl_). The degree of conversion, cytotoxicity, softening in solvent, contact angle and free surface energy, antibacterial activity, shear bond strength, and mineral deposition were evaluated. Adhesives achieved degree of conversion higher than 50% and cell viability higher than 90%. G_BNNT_ and G_ATAB_ adhesives exhibited reduced softening in solvent. Mean free surface energy was decreased in the G_BNNT_ adhesive. Significant reduction in bacterial growth was observed in the G_BNNT/ATAB_. No statistical difference was found for shear bond strength. Mineral deposition was found in G_BNNT_, G_ATAB_, and G_BNNT/ATAB_ groups after 14 and 28 days. The addition of 0.2% BNNT/ATAB to an experimental orthodontic adhesive inhibited bacterial growth and induced mineral deposition without affecting the properties of the material.

## 1. Introduction

Fixed orthodontic appliances act as biofilm retentive factors that make oral hygiene difficult for patients [1]. The lack of adequate oral hygiene results in alterations of the oral microbial flora [2] and a consequent decrease in salivary pH [1,2]. Thus, fixed orthodontic appliances make patients susceptible to the accumulation of bacterial plaque around the brackets, which leads to enamel demineralization in the form of white spot lesions [3]. Sundararaj [1] reported a prevalence of white spot lesions in patients with fixed orthodontic appliances of 68.4%, which indicates the need for new preventive methods to reduce the occurrence of these lesions [1].

Orthodontic materials with antimicrobial and bioactive activities are desirable [4], and could be an alternative to prevent white lesions. The addition of antimicrobial agents [5] has been shown to inhibit or reduce bacterial growth [6] and to induce the remineralization of enamel that is already affected [7]. Alkyl trimethyl ammonium bromide (ATAB) is a quaternary ammonium compound that is considered a surfactant and widely used as a disinfectant and a preservative in pharmaceuticals and in the food industry [8]. The antimicrobial action of ATAB has been described in the literature since 1985 [9], and small concentrations are sufficient to inhibit bacterial growth [8], including the growth of both gram-positive and gram-negative bacteria. ATAB also effectively inhibits yeast and fungi growth [9,10]. In dentistry, quaternary ammonium compounds are used in adhesive resins to promote antimicrobial activity [4,10,11,12] and to decrease bacterial attachment in the enamel and biofilm accumulation [11]. However, to date, there are no dentistry studies of the use of alkyl trimethyl ammonium bromide. 

The addition of an antimicrobial agent may decrease the properties and biocompatibilities of materials [10]. With the aim of improving the mechanical properties of materials and inducing mineral deposition, tubular nanoparticles are being used as fillers in resin-based materials [13]. Boron nitride nanotubes (BNNTs) are tubular nanoparticles, with properties similar to those of graphene [14], that have characteristics that include biocompatibility, chemical stability, superhydrophobicity, oxidation resistance, and thermal and electrical insulation [15,16,17]. BNNTs also have the ability to induce the formation of hydroxyapatite [18], which can elicit a remineralizing action in white spot lesions. Because BNNT functionalized nanoparticles may increase the mechanical resistance of materials [17], and they have the abilities to carry drugs [16] and the potential to confer antibacterial activities to materials [19], it is possible to functionalize particles of boron nitride nanotubes with alkyl trimethyl ammonium bromide for use as fillers in orthodontic adhesives. 

The development of an experimental orthodontic adhesive with potential antimicrobial action could represent a possible alternative for the bonding of brackets the control of the development of white spot lesions. The aim of this study is to develop and characterize an experimental orthodontic adhesive that incorporates boron nitride nanotubes and alkyl trimethyl ammonium bromide as a filler.

## 2. Materials and Methods 

### 2.1. Experimental Adhesive Resin Formulation

The experimental orthodontic adhesives were formulated with a mixture of 75 wt.% bisphenol A glycidyl methacrylate (Bis-GMA) and 25 wt.% triethylene glycol dimethacrylate (TEGDMA). To enable a photoinitiator system, 1 mol% each of camphorquinone (CQ), ethyl-4-dimethylamino benzoate (EDAB), and diphenyliodonium hexafluorophosphate (DPIH) were added, and 0.01 wt.% butylated hydroxytoluene (BHT) was added as a polymerization inhibitor. All of these agents were from Sigma-Aldrich (St. Louis, MO, USA). To adjust the viscosity, 5 wt.% of fumed silica (AEROSIL 200; Evonik, Piscataway, NJ, USA) [5] was added.

Boron nitride particles (BNNT, LLC, Newport News, VA, USA) and alkyl trimethyl ammonium bromide (Sigma-Aldrich, St. Louis, MO, USA) were dissolved in 10 mL of absolute alcohol at a ratio of 1:1. The mixture was maintained under ultrasonic agitation for 3 h and subsequently maintained for 7 days in a desiccator at 37 °C for total evaporation of the solvent. The following agents were added to the experimental orthodontic adhesives: 0.2 wt.% BNNT and alkyl trimethyl ammonium bromide (ATAB) (G_BNNT/ATAB_); 0.1 wt.% BNNT (G_BNNT_); 0.1 wt.% of ATAB (G_ATAB_); and the addition of the filler was omitted in one group (G_Ctrl_). Thus, there were a total of four experimental groups.

### 2.2. Degree of Conversion

The degree of conversion (DC) was measured by Fourier transform infrared spectroscopy (FTIR) on a spectrometer (Vertex 70, Bruker Optics, Ettlingen, Germany) equipped with an attenuated total reflectance device (Platinum ATR-QL, Bruker Optics, Ettlingen, Germany) with a horizontal diamond crystal. The adhesives were inserted directly onto the crystal in a 6-mm diameter and 2-mm-thick polyvinylsiloxane matrix (ADSIL, VIGODENT; Rio de Janeiro, Brazil) (n = 3). The samples were photoactivated for 20 s using LED equipment (Radii, SDI, Bayswater, Australia) with irradiation of 1200 mW/cm^2^ [6]. The absorbance spectra were obtained before and after sample polymerization. DC was calculated as described in a previous study [7]. 

### 2.3. Cytotoxicity

For the cytotoxicity assay, human keratinocytes of the HaCaT lineage were used. The cell line was obtained from a cell bank (Banco de Células do Rio de Janeiro, Rio de Janeiro, Brazil). The cells were cultivated in Dulbecco’s modified Eagle medium (DMEM), supplemented with 10% fetal bovine serum and 1% penicillin in 37 °C with 5% of CO_2_. Eluates were prepared by immersing samples (3-mm diameter × 1-mm thickness) from each experimental group (n = 3) in 1 mL DMEM for 24 h. The cells were later placed into 96-well plates at a concentration of 5 × 10^3^ and treated with 100 μL of an eluate. After 72 h, the cells were fixed with 10% trichloroacetic acid (TCA), incubated at 4 °C for one hour, washed six times with running water and dried at room temperature. Four percent sulforhodamine B (SRB, Sigma-Aldrich, St. Louis, MO, USA) was added to color the cells, and the plate was incubated for 30 min at room temperature. The plates were then washed four times with 1% acetic acid to remove the excess unbound dye and dried at room temperature. Trizma solution was used to resuspend the cells, and the microplates were measured at 570 nm (Multiskan EX Microplate Reader, MTX Lab Systems, Vienna, Austria) [20].

### 2.4. Softening in Solvent 

To evaluate the softening in solvent, five specimens from each group were analyzed. The specimens were made with a polyvinyl siloxane matrix with a 6.0-mm (±0.5 mm) diameter and 1.0-mm (±0.2 mm) thickness and photoactivated with a LED (Radii Cal., SDI Ltd., Melbourne, Australia) for 20 s on each side. Subsequently, the specimens were polished before Knoop microhardness (KHN) measurements were performed. Three indentations made with a 10-g load were created over 5 s at distances equal to 100 μm using a microdurometer (HMV 2, Shimadzu, Japan), before (KHN1) and after immersion in a solution of 50% ethanol and 50% water for 2 h (KHN2). The percentage of variation in the Knoop hardness (ΔKNH%) was calculated for each specimen [13].

### 2.5. Contact Angle and Free Surface Energy

The contact angle analysis was performed by the sessile drop method. Forty discs of 6 mm in diameter and 1 mm in thickness were photoactivated for 20 s on each side with a LED (Radii Cal, SDI Ltda.,Victoria, Australia), embedded in acrylic resin and divided into 5 groups according to each concentration of the inorganic filler (n = 10). Subsequently, polishing was performed on a rotary electric polishing machine with multiple polishing systems to obtain a flat surface (Model 3v, Arotec, Cotia, Brazil). The contact angle measurements were performed with an optical tensiometer (Theta, Attension Biolin Scientific, Stockholm, Sweden) by imaging the distributions of a polar distilled water liquid (n = 5) and non-polar alpha-bromonaphthalene liquid (n = 5) on the sample surface. Three replicates per liquid were performed for each specimen. The images were captured for 20 s, and the contact angles were measured after 10 s of contact of the droplet with the surface of the disc. The average between the right and left angles was calculated according to the point of the liquid–air–tooth intersection. The Energy Surface Free Energy calculations were performed according to the OWRK/Fowkes method using the OneAttension program (Biolin Scientific, Stockholm, Sweden), and the results were reported in mN/m [13].

### 2.6. Antibacterial Activity

For the antibacterial activity assay, three specimens per group with 6-mm (±0.1 mm) diameters and 1.0-mm (±0.01 mm) thicknesses were fixed in the lid of a 48-well plate and submitted to hydrogen peroxide sterilization. Each well of the test plate contained 900 μL of brain–heart infusion broth (BHI; Aldrich Chemical Co., St. Louis, MO, USA) with 1% sucrose and 100 μL of a suspension of Streptococcus mutans (NCTC 10449), which corresponded to 6.66 (±0.05) log CFU/mL. Additionally, 3 wells without the specimens containing 900 μL of BHI broth were inoculated with 100 μL of the bacterial suspension as controls. For the evaluation of antibacterial activity against biofilm formation on the resin surface, the specimens were removed from the lid, vortexed for 1 min in 1 mL of saline solution (0.9%) and diluted until a concentration of 10^−6^ was reached. For the planktonic bacteria viability evaluation, 100 μL from each well was diluted in 900 μL of saline solution until a 10^−6^ dilution was reached. Two 25-μL drops of each dilution were plated in BHI agar in Petri dishes, and the dishes were incubated for 48 h at 37 °C. The numbers of colony forming units (CFUs) were counted using microscopy and transformed to log UFC/mL values [6].

### 2.7. Shear Bond Strength 

Sixty pre-molar teeth were used to evaluate the shear bond strength (n = 15). The teeth were fixed with acrylic resin using a metal matrix of 15 mm in height and 21 mm in diameter with the vestibular face perpendicular to the acrylic base. The labial surfaces of the teeth were preconditioned with 37% phosphoric acid for a period of 30 s, rinsed with water for 30 s and air-dried. Brackets (Roth Max, Morelli, Sorocaba, Brazil) were fixed to the centers of the vestibular faces of the teeth using the experimental orthodontic adhesives (G_Ctrl_, G_BNNT_, G_ATAB_ and G_BNNT/ATAB_). A 300-g force was applied to the surface of the brackets to standardize the adhesive thickness, and the excess adhesive was removed. The adhesive was photoactivated for 10 s on each face of the bracket; thus, the photoactivation totaled 40 s. The samples were subjected to the shear bond strength test in a universal test machine (Shimadzu EZ Test EZ-SX, Kyoto, Japan). Using a knife-edge chisel (0.1 mm) applied at 180° to the labial face of the tooth and positioned at the adhesive-enamel interface, a force was applied with the speed of 1 mm/min until the moment the bracket was detached, and the results were recorded in MPa. After the shear bond strength test, the residual adhesive on the tooth surface (Adhesive Remnant Index, ARI) was evaluated with a stereomicroscope (×10, SZX16, Olympus, Center Valley, PA, USA) as previously described [21].

### 2.8. Mineral Deposition

For the evaluation of mineral deposition, three disks of 6 mm (±0.1 mm) in diameter and 1 mm (±0.1 mm) in thickness were made from each adhesive. The surfaces of the specimens were evaluated before and after immersion in 10 mL of simulated body fluid (SBF) at 37 °C for 14 days and 28 days by means of Raman spectroscopy (Senterra, Bruker Inc., Karlshure, Germany) using a 785-nm laser. The SBF was prepared as previously described. The standard area (2000 μm × 2000 μm) was analyzed (900 equidistant points) for each specimen. Changes in the intensities of the peaks located in the regions of the 960 cm^−1^ wavenumbers (characteristic of the bonds present in PO43 ions) were taken as references for the formation of hydroxyapatite in the Raman spectra [7].

### 2.9. Statistical Analysis

The normality of the data was analyzed using the Shapiro-Wilk test. The KHN1 and KHN1 data regarding degradation in solvent were normally distributed and compared with a paired Student’s *t*-test. One-way Analysis of Variance were applied to examine the contact angle, free surface energy, degree of conversion, cytotoxicity, antimicrobial test results, KHN1 and ΔKNH% degradation in the solvent and shear strength test results. When differences between the groups were identified, Tukey’s multiple comparison tests were applied. All analyses were performed adopting a significance level of 5% using appropriate statistical software.

## 3. Results

**Figure 1:**
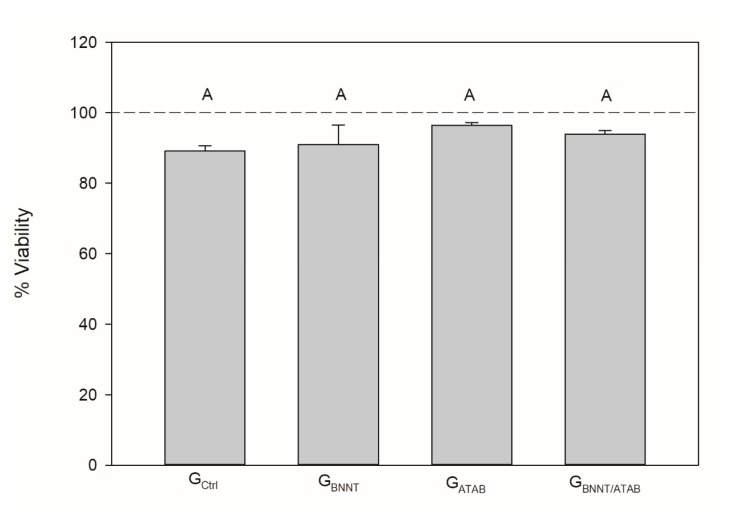
Mean (±standard deviation) of Epithelial cells viability (%). Distinct capital letters indicate a significant difference (*p* < 0.05) between groups. The reference line represents 100% cell viability as observed in wells without treatment.

**Figure 2:**
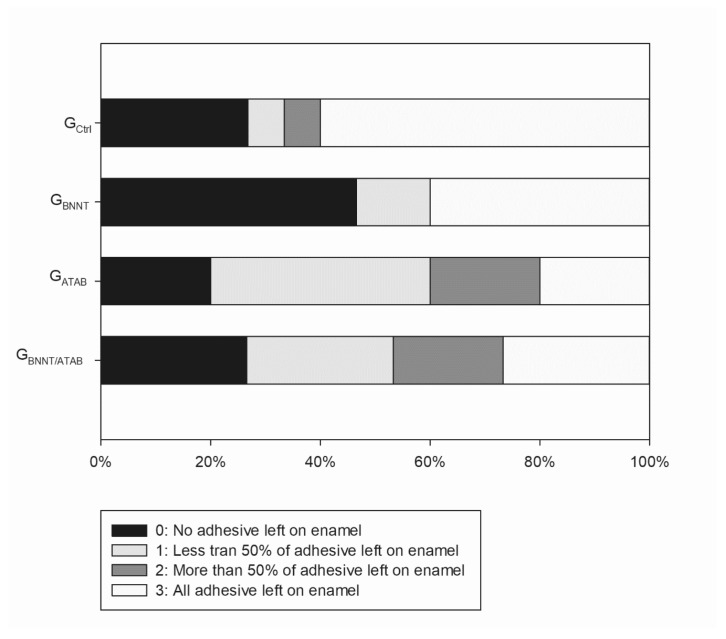
Adhesive Remnant Index (ARI) scores for shear bond strength test.

**Figure 3:**
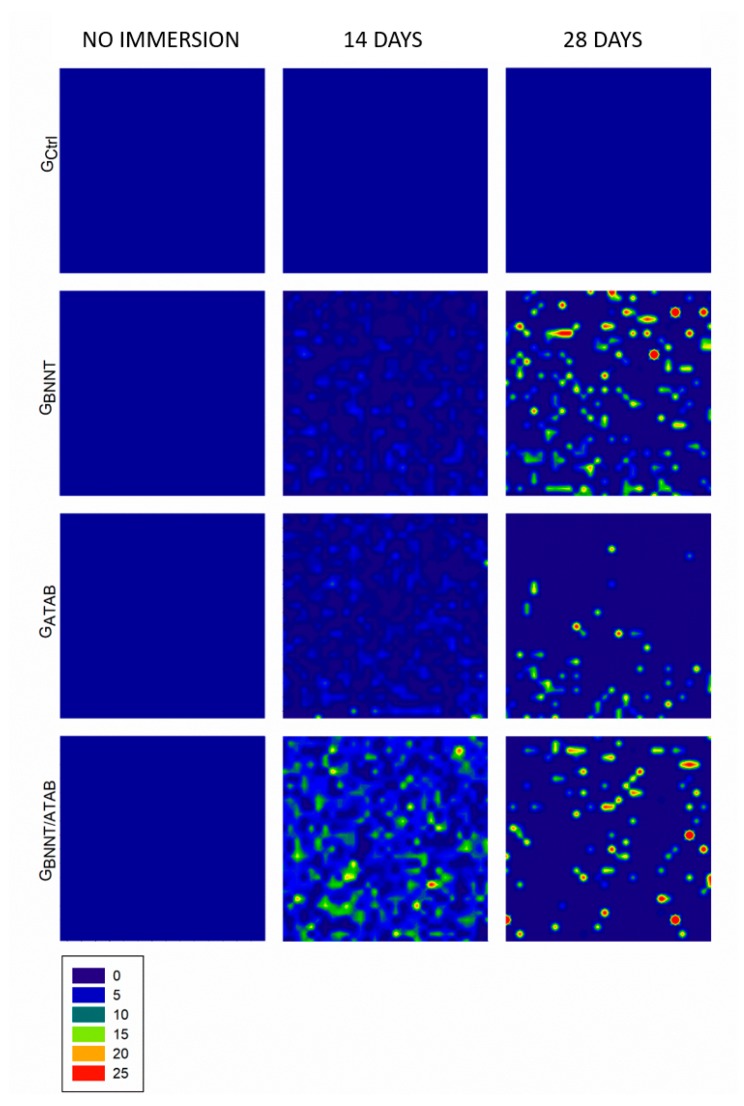
Representative image of mineral deposition test from 960 cm^−1^ Raman peak. The blue areas are indicative of absence of mineral deposition. The intensity of 960 cm^−1^ peak is represented by different colors from green to red.

The mean degree of conversion and softening in solvent values are presented in Table 1. The percentage of converted monomers was higher for G_ATAB_ (58.98 ± 0.51; *p* < 0.05) when compared to the other tested groups. The G_Ctrl_ (56.36 ± 0.82) and G_BNNT/ATAB_ (55.64 ± 1.15) groups presented no statistical difference between DC results (*p* > 0.05). G_BNNT_ (52.64 ± 0.40) exhibited the lowest DC (*p* < 0.05) in this study. Regarding softening in the solvent, the initial microhardness values (KNH1) were similar for all groups (*p* > 0.05; Table 1). The values after immersion in ethanol were lower than the initial values in all groups (*p* < 0.05). The G_BNNT_ and G_ATAB_ groups presented less softening than the G_Ctrl_ and G_BNNT/ATAB_ groups. This was found as the percentage differences between KHN1 and KHN2 (ΔKHN%) were lower for the G_BNNT_ (12.74 ± 7.32) and G_ATAB_ (13.67 ± 8.28) compared with the G_Ctrl_ (34.29 ± 9.18). No significant reduction in cell viability was observed for the experimental orthodontic adhesives and the control group. All groups presented cell viabilities higher than 90%, as illustrated in Figure 1, and the addition of BNNT and/or ATAB did not compromise the percentage of viable cells. The contact angle and surface free energy results are expressed in Table 2. The contact angle of water above samples was significantly higher for G_BNNT_ group (*p* < 0.05). When alpha-bromonaphthalene was used, the BNNT-containing groups (G_BNNT_ and G_BNNT/ATAB_) presented higher contact angle values. These values were used to calculate the Surface Free Energy, which is found in Table 2, and decreased values were found for the G_BNNT_ (*p* < 0.05; Table 2). The results of the antibacterial activity tests are presented in Table 3. A significant reduction in bacterial growth was observed in the G_BNNT/ATAB_ group after 24 h of incubation at 37 °C (*p* < 0.05) in the biofilm analysis, that is, in the bacteria that adhered in the samples during the test. The planktonic analysis did not reveal any significant differences. No statistical difference was found for shear bond strength (Table 3) for all of the experimental orthodontic adhesives (*p* > 0.05). The adhesive remnant index (ARI scores; Figure 2) were mainly scored as 3 for G_Ctrl_, indicating that the adhesives remained in the enamel after bracket debonding. G_BNNT_ and G_BNNT/ATAB_ scores were mainly 0, and the G_ATAB_ scores were mainly 1, and less or no adhesive remnant was found in the enamel. The mineral deposition is found in Figure 3, and the different colors indicate different intensities of the phosphate peak (960 cm^−1^) in the orthodontic adhesives. Phosphate deposition was found in the G_BNNT_, G_ATAB_, and G_BNNT/ATAB_ groups after 14 and 28 days of immersion in SBF, as observed for the higher peak intensities in blue to orange colors in Figure 1. 

## 4. Discussion

The formation of white spot lesions around brackets is a common complication of fixed orthodontic treatments. In the present study, experimental orthodontic adhesives that included particles of boron nitride nanotubes and alkyl trimethyl ammonium bromide were formulated. These adhesives were produced with the objective of inhibiting or reducing bacterial growth and inducing the remineralization of already affected enamel without affecting the mechanical properties of the material. 

The degree of conversion (DC) of an orthodontic adhesive influences the mechanical properties of the adhesive due to the cross-linking density that is determined during polymerization [22]. In this study, the G_BNNT_ adhesive presented with the lowest degree of conversion among all groups. According to our results, when added to an adhesive resin at levels above 0.1 wt.%, BNNT resulted in a decrease in the degree of conversion [23]. BNNTs originate from graphene and have a grayish color before polymerization [14]; thus, the availability of light in the resin matrix is decreased, which leads to a lower DC of the adhesive resin. 

A low degree of conversion of adhesives may cause biological reactions because it enables the release of unconverted monomers. Quaternary ammonium compounds exhibit low cytotoxicity [10]; however, ATAB is an unpolymerizable monomer, and it is cytotoxic to keratinocytes. Cytotoxicity was examined using keratinocytes, because keratinocyte cells comprise most of the oral mucosa [24]. To date, there are no reports available on the adverse effects of BNNT on living cells [23,25]. Despite the differences in the degrees of conversion found between the tested adhesives, no significant differences in cytotoxicity were found for any of the specimens compared to the control, and the cell viabilities of the keratinocytes were above 90% for all of the adhesives.

One method of studying the stability of a polymer network is through softening in solvent [26]. The softening in solvent after 2 h of immersion in alcohol was lower in the groups that contained G_BNNT_ and G_ATAB_ than in the GCtrl group. BNNT has a high modulus of elasticity [18] and is considered to be a superhydrophobic material [16]; thus, this material reduces the degradation of adhesive resins over time and thus increases the resistance to hydrolytic degradation [27]. 

BNNTs may reach contact angles above 170° with water [11]. The incorporation of BNNTs into orthodontic adhesives produced adhesives with higher contact angles and lower free surface energies, compared with the other experimental orthodontic adhesives. ATAB is considered to be a surfactant [8], and when added to adhesives with BNNT (G_BNNT/ATAB_), ATAB increased the hydrophilicity of the material, which resulted in an increase in the surface free energy (59.41 ± 4.19 mN/m ) compared to the G_BNNT_ adhesive (44.86 ± 3.41 mN/m). The low free surface energy of GBNNT decreased the wetting of the orthodontic adhesive and thus decreased bacterial fixation around the brackets [28]. 

Furthermore, BNNT particle size ranges from 5 to 10 nm according to previous studies [29]. The addition of nanoparticles to adhesives is capable of conferring antimicrobial activity due to the particle size and large free surface area [30]. The antimicrobial activity of ATAB has previously been described in the literature [8,9,10] and is related to the long alkyl chain length. However, the separate additions of 0.1% BNNT and 0.1% ATAB were insufficient to ensure antimicrobial activity. In contrast, when 0.2% BNNT/ATAB was added, a decrease in bacterial adhesion to the specimens was observed. This observation can be explained by a synergistic effect of the two fillers and by the addition of a double-nano filler. The planktonic analysis revealed no significant differences of any of the groups, compared with the broth without the specimens, in terms of the growth of bacteria in the medium over 24 h. These data indicate that the specimens did not leach or did not leach enough to decrease the growth of bacteria in the medium, which may lead to a maintenance of the contact-dependent antibacterial activity over time. Despite the similar bacterial growths, lower bacterial adhesion was observed in the test specimens, which indicates that the novel adhesives did not leach, but were rather antimicrobial by contact. 

The shear bond strength data did not reveal any differences between the experimental groups and the control group. To regulate the viscosities of the materials, 5 wt.% silica was added to the experimental adhesives. When an orthodontic adhesive is viscous, some researchers have chosen to use a more fluid resin as a primer prior to the application of the orthodontic adhesive to improve the imbrication between the adhesive and the enamel [31]. The addition of BNNT may increase the mechanical strength of a material [23], and in our study, we did not use any primer prior to the application of the orthodontic adhesive and did not observe any effects on shear strength. The use of a primer may be omitted for any type of orthodontic adhesive, because no differences in the shear strengths of bonded brackets result from the use of a primer [31].

We need new materials with bioactive fillers [4] that are capable of inducing the deposition of phosphate, and this study found mineral deposition in all of the experimental groups after 14 and 28 days of immersion in SBF, as observed based on the 960 cm^−1^ Raman peak (Figure 3). The immersion of specimens in SBF is a fast and reliable method for predicting the in vitro bioactivities of new biomaterials [32]. SBF is the most frequently used solution to address the in vitro remineralization ability of different materials and was used here to the evaluation of the adhesive behavior when in contact with ionic solutions in an aqueous environment. The mineral deposition capacity of BNNT has previously been demonstrated [18]. Lahiri [18] observed an initial change in the peak for BNNTs (1357 to 1363 cm^−1^), after seven days of immersion in SBF, that was due to the aging of the BNNTs in the SBF, and this effect did not continue to progress with longer immersion times [18]. Despite the aging of the BNNTs, due to contact with the SBF, there was an increase in the phosphate deposition between days 14 and 28, which contrasts with the observations following the addition of BNNT/ATAB. The mineral deposition due to ATAB indicated that phosphate deposition occurred after 14 days of immersion in SBF, and this deposition decreased after 28 days, which may have occurred due to leaching of the ATAB after 14 days of immersion in SBF.

## 5. Conclusions

In the present study, BNNTs and ATAB were successfully incorporated into an experimental orthodontic adhesive for the investigation of the antibacterial and remineralizing properties of these materials. The addition of 0.2% BNNT/ATAB to an experimental orthodontic adhesive inhibited bacterial growth and induced mineral deposition without affecting the properties of the material.

## Figures and Tables

**Table 1:** Mean and standard deviation of degree of conversion (DC) and microhardness value of the model adhesives before (KHN1), after immersion in solvent (KHN2), and the variation of microhardness values [∆%]. The different capital letters in the different columns indicate statistical difference between groups (*p* < 0.05). The different small letters indicate statistical difference (*p* < 0.05) between the initial (KHN1) and the final (KHN2) measurements.

Groups	DC [%]	KHN1	KHN2	∆KHN%
G_Ctrl_	56.36 (±0.82) B	25.89 (±2.71) Aa	16.82 (±0.84) b	34.29 (±9.18) A
G_BNNT_	52.64 (±0.40) C	23.92 (±1.55) Aa	20.82 (±1.41) b	12.74 (±7.32) B
G_ATAB_	58.98 (±0.51) A	23.14 (±2.15) Aa	19.84 (±0.62) b	13.67 (±8.28) B
G_BNNT/ATAB_	55.64 (±1.15) B	23.90 (±2.99) Aa	18.99 (±2.73) b	20.43 (±10.98) AB

**Table 2:** Outcomes obtained for the contact angle and surface free energy with means and standard deviation for each resin tested. The different capital letters in the different columns indicate statistical difference between groups (*p* < 0.05).

Groups	Contact Angle (θ, °)		SFE (mN/m)
	Water	α-Br	
**G_Ctrl_**	52.48 (±11.72) B	12.39 (±5.39) C	58.54 (±6.10) A
**G_BNNT_**	70.25 (±8.58) A	36.96 (±4.12) A	44.86 (±3.41) B
**G_ATAB_**	51.81 (±7.28) B	23.29 (±3.65) B	57.46 (±3.89) A
**G_BNNT/ATAB_**	46.85 (±5.87) B	37.80 (±2.69) A	59.41 (±4.19) A

**Table 3:** Mean and standard deviation of antibacterial activity for the biofilm, planktonic evaluation (log CFU/mL), and shear bond strength (n = 15) of experimental orthodontic adhesives (MPa). The different capital letters in the different columns indicate statistical difference between groups (*p* < 0.05).

Groups	Biofilm Evaluation (log CFU/mL)	Planktonic Evaluation (log CFU/mL)	Shear Bond Strength (MPa)
G_Ctrl_	5.94 (±0.26) A	8.21 (±0.07) A	12.37 (±3.01) A
G_BNNT_	5.79 (±0.11) A	8.19 (±0.08) A	14.17 (±3.39) A
G_ATAB_	5.78 (±0.16) A	8.19 (±0.06) A	13.62 (±1.64) A
G_BNNT/ATAB_	5.14 (±0.10) B	8.22 (±0.08) A	13.22 (±3.05) A
Planktonic control	-	8.18 (±0.06) A	-
